# Performance of Antigen-Based Testing as Frontline Diagnosis of Symptomatic COVID-19

**DOI:** 10.3390/medicina57080852

**Published:** 2021-08-21

**Authors:** Efrén Murillo-Zamora, Xóchitl Trujillo, Miguel Huerta, Mónica Ríos-Silva, Oliver Mendoza-Cano

**Affiliations:** 1 Departamento de Epidemiología, Unidad de Medicina Familiar No. 19, Instituto Mexicano del Seguro Social, Av. Javier Mina 301, Col. Centro, Colima C.P. 28000, Mexico; efren.murilloza@imss.gob.mx; 2 Facultad de Medicina, Universidad de Colima, Av. Universidad 333, Col. Las Víboras, Colima C.P. 28040, Mexico; 3 Centro Universitario de Investigaciones Biomédicas, Universidad de Colima, Av. 25 de julio 965, Col. Villas San Sebastián, Colima C.P. 28045, Mexico; rosio@ucol.mx (X.T.); huertam@ucol.mx (M.H.); 4 Centro Universitario de Investigaciones Biomédicas, Universidad de Colima-Cátedras CONACyT, Av. 25 de julio 965, Col. Villas San Sebastián, Colima C.P. 28045, Mexico; mrios@ucol.mx; 5 Facultad de Ingeniería Civil, Universidad de Colima, km. 9 Carretera Colima-Coquimatlán, Coquimatlán, Colima C.P. 28400, Mexico

**Keywords:** COVID-19, SARS-CoV-2, immunologic tests

## Abstract

*Background and Objectives*: To evaluate the performance of antigen-based detection tests as the frontline diagnosis of coronavirus disease 2019 (COVID-19). *Materials and Methods*: We conducted a nationwide retrospective cohort study in Mexico. A cross-sectional analysis of a cohort study was conducted in Mexico and data from 15,408 suspected (all of them symptomatic) cases of COVID-19 were analyzed. The results of antigen-based tests were compared with those obtained by molecular (polymerase chain reaction-based) assays. *Results*: The antigen-based tests showed sensitivity below 50% and high specificity in all the analyzed age groups. The highest Youden index (J) was observed among adults aged 25–44 years old (45.5, 95% CI 43.7–47.3). *Conclusions*: We documented the poor performance of serologic techniques as frontline diagnosis of symptomatic COVID-19 and inaccurate results may impact negatively on pandemic progression.

## 1. Introduction

The burden of the coronavirus disease 2019 (COVID-19) caused by severe acute respiratory syndrome Coronavirus 2 (SARS-CoV-2) has been high in Mexico. By the end of January 2021, about 1.8 million laboratory-confirmed cases of COVID-19 had been registered together with 150 thousand associated deaths [1].

Since the beginning of the pandemic, nucleic acid amplification tests (NAATs) had been used for the clinical diagnosis of COVID-19 in Mexican public healthcare settings. However, given that specialized laboratory techniques are required, clinical samples are commonly shipped to centralized facilities, causing delays in result reporting and lowering the impact of clinical decision-making and transmission interruption [2].

Antigen-based tests are immunoassays that represent an inexpensive and easy-to-use alternative in COVID-19 diagnosis and were widely implemented in December 2020 in the public healthcare system of Mexico. By the end of the first trimester of 2021, 12 commercial tests had been approved to be used in the Mexican territory: SARS-CoV-2 Antigen Assay Kit (Zybio Inc., Shenzhen, China); Panbio™ COVID-19 Ag Rapid Test Device (Nasal; Abbott Laboratories, Chicago, IL, USA); ECOTEST COVID-19 Antigen Rapid Test Device (Assure Tech (Hangzhou) Co., Hangzhou, China); 2019-NCoV Antigen Test Kit (Genrui Biotech Inc., Shenzhen, China); COVI-STIX™ COVID-19 Virus Rapid Antigen Detection Test (ZhengZhou Fortune Bioscience Co., Zhengzhou, China); COVID-19 Antigen Rapid Test Cassette (Hangzhou Clongene Biotech Co., Yuhang, China); CerTest SARS-CoV-2 Ag Test (Certest Biotec SL., Zaragoza, Spain); GeneFinder™ COVID-19 Ag Rapid Test (Osang Healthcare Co., Anyangcheondong-ro, Anyang, Korea); SARS-CoV-2 Antigen Test Kit (Genrui Biotech Inc., Shenzhen, China); SARS-CoV2 Antigen Rapid Test System (Monocent Inc., Chatsworth, CA, USA); SARS-CoV-2 Rapid Antigen Test (SD BioSensor Inc., Suwon, Korea); STANDARD™ Q COVID-19 Ag Test (SD BioSensor Inc., Suwon, Korea); Panbio™ COVID-19 Ag Rapid Test Device (Abbott Rapid Diagnostics Jena GmbH, Jena, Germany); and Sofia2 SARS Antigen FIA (Quidel Corporation, San Diego, CA, USA) [3]. 

We aimed to assess the performance of rapid antigen detection tests as the frontline diagnosis of COVID-19 in comparison to molecular techniques in a real-world scenario.

## 2. Materials and Methods

We conducted a cross-sectional analysis of a cohort study that took place in Mexico. Patients fulfilling suspected COVID-19 criteria and disease onset from December 2020 to January 2021, and in whom antigen-based detection tests and NAATs (reverse transcription-polymerase chain reaction, RT-PCR) were performed in nasopharyngeal swab specimens, were analyzed. Only symptomatic patients were eligible. Subjects with 2 or more days elapsed between both clinical specimens being taken were excluded. A wider description of research methods from the follow-up study was previously published [4,5].

The molecular assays were carried out by using the SuperScript III Platinum One-step RT-PCR System (Invitrogen, Carlsbad, CA, USA; catalog: 12574035) in the 7500 Fast Real-Time PCR System (Applied Biosystems, Foster City, CA, USA). The employed tests were any of the kits approved to be used in Mexico, since we were unable to identify which of them was used in each enrolled patient.

The performance of the antigen-based test was evaluated in terms of sensitivity, specificity, accuracy, and positive and negative likelihood ratio (LR+/−). Age-stratified (0–13; 14–17; 18–24; 25–44; 45–79 and 80 years or above) estimators were obtained. Area Under Receiver Operating Characteristic curves (AUROCs), Youden’s index (J), and 95% confidence intervals (CI) were also computed. The analytical procedure was performed by using the Stata Statistical Software: Release 15 (StataCorp LLC; College Station, TX, USA). This study was approved by the Local Ethics in Health Research Committee (601) of the *Mexican Institute of Social Security* (R-2021-601-022).

## 3. Results

Data from 15,408 individuals were analyzed. A total of 49.5% of participants were female and their mean age (±standard deviation) was 47.2 ± 18.5 years. The mean days elapsed from symptoms onset to clinical sampling were 3.4 ± 1.3 and 3.3 ± 1.3 for RT-PCR and antigen-based testing, respectively. From patients with a positive RT-PCR (*n* = 8653), 3937 and 4710 had positive and negative antigen-based results, respectively. Additionally, among patients with a negative molecular-based result (*n* = 6755), 297 had a positive antigen-based result and the rest of them also were discarded by rapid testing.

The performance estimates are presented in Table 1. The sensitivity of rapid tests in diagnosing COVID-19 was low, and the mean estimate was 45.5% (95% CI 44.7–46.3%); it was lower among children (35.9%, 95% CI 30.9–40.9%) and teenagers (37.1%, 95% CI 27.2–47.4%) when compared with older patients. The age-stratified specificity went from 94.5 (95% CI 94.0–95.0, 45–79 years old) to 99.3 (97.6–99.8, 0–13 years old).

## 4. Discussion

During the COVID-19 pandemic, antigen-based testing has played a major role in limiting viral pathogen spread and medical management of infected patients. However, our findings suggest that about 6 out of 10 patients with SARS-CoV-2 infection may have a negative antigen-based test result. Inaccurate results may negatively impact on clinical management of patients and favor viral spread.

The overall sensitivity from our study was higher than estimated in a previously published study that took place in Belgium (45.5% vs. 30.2%, *p* = 0.002) [6]. The authors from the European study do not specify if asymptomatic patients were screened and if that occurred and given that we only analyzed patients with suggestive symptoms of COVID-19, it may partially explain these discrepant findings.

Factors affecting SARS-CoV-2 testing are numerous and include, among others, the viral load and integrity of specimen collection and handling. In our analysis, the test sensitivity among children (35.9, 95% CI 30.9–40.9) and teenagers (37.1, 95% CI 27.2–47.4) was below the mean; we hypothesize that a higher rate of imperfectly generated specimens among younger subjects may at least partially account for the bias observed. Users should follow the manufacturer’s instructions, as well as state and local guidance, for when and how often to perform testing on control specimens [7].

We were unable to determine which specific diagnostic kit was used in each participant and that represents a limitation of our analysis. According to normative standards, the rapid-antigen tests authorized to be employed in the diagnosis of COVID-19 in healthcare settings from Mexico must have sensitivity and specificity above 80% and 97%, as corresponding [8].

## 5. Conclusions

Antigen-based tests are relevant to reduce the COVID-19 pandemic’s progression. However, their performance in the study sample was below the desired standard, mainly in terms of sensitivity. Efforts focusing on assuring the quality of clinical samplings and their handling are needed to provide an accurate and opportune diagnosis of SARS-CoV-2 infection.

## Figures and Tables

**Table 1:** Performance of antigen-based tests in the diagnosis of coronavirus disease 2019, Mexico 2020–2021.

Age Group (Years)	*n*	Prevalence (%)	Indicator (95% Confidence Interval, CI)					Likelihood Ratio (LR)	
			Sensitivity (%)	Specificity (%)	Accuracy (%)	Receiver Operating Characteristic Area	Youden’s Index (J)	+	−
0–13	366	6.8	35.9 (30.9–40.9)	99.3 (97.6–99.8)	88.3 (84.5–91.4)	0.676 (0.617–0.736)	35.2 (28.5–40.7)	54.266	0.645
14–17	95	15.8	37.1 (27.2–47.4)	96.7 (91.1–99.3)	74.7 (64.8–83.1)	0.669 (0.585–0.753)	33.8 (18.3–46.7)	11.142	0.650
18–24	908	20.3	46.6 (43.3–49.9)	98.0 (96.9–98.8)	77.0 (74.1–76.7)	0.723 (0.697–0.749)	44.6 (40.2–48.7)	22.764	0.545
25–44	5936	25.6	49.8 (48.5–51.1)	95.7 (95.2–96.2)	74.2 (73.1–75.3)	0.727 (0.717–0.737)	45.5 (43.7–47.3)	11.538	0.525
45–79	7426	30.6	43.1 (42.0–44.2)	94.5 (94.0–95.0)	60.2 (59.1–61.3)	0.688 (0.680–0.696)	37.6 (36.0–39.2)	7.820	0.603
80+	677	32.2	47.0 (43.2–50.8)	95.7 (93.9–97.1)	63.8 (60.1–67.4)	0.713 (0.687–0.740)	42.7 (37.1–47.9)	10.987	0.554
All	15,408	27.5	45.5 (44.7–46.3)	95.6 (95.3–95.9)	67.5 (66.8–68.2)	0.706 (0.700–0.711)	41.1 (40.0–42.2)	10.348	0.570

The overall accuracy and AUROC were 67.5 (95% CI 66.8–68.2) and 0.706 (95% CI 0.700–0.711), respectively. The age-stratified AUROCs were significantly different in all the analyzed age groups (*p* < 0.001). The highest J (45.5, 95% CI 43.7–47.3) was observed in adults aged 25–44 years old. The LR+ and LR− were 10.348 and 0.570, respectively.

## Data Availability

The datasets generated during and/or analyzed during the current study are available from the corresponding author on reasonable request.
